# Supplementary material for: Association between oral microbiome and seven types of cancers in East Asian population: a two-sample Mendelian randomization analysis
Source: Front Mol Biosci. 2023 Nov 21;10:1327893. doi: 10.3389/fmolb.2023.1327893 (PMC10702768; doi:10.3389/fmolb.2023.1327893)
Supplement: Supplementary file 1 [file DataSheet1.ZIP › Supplementary Materials/MR plots for tongue/tongue═╝/Ovarian cancer/pheno.154_to_ovarian cancer_leave_one_out.pdf]

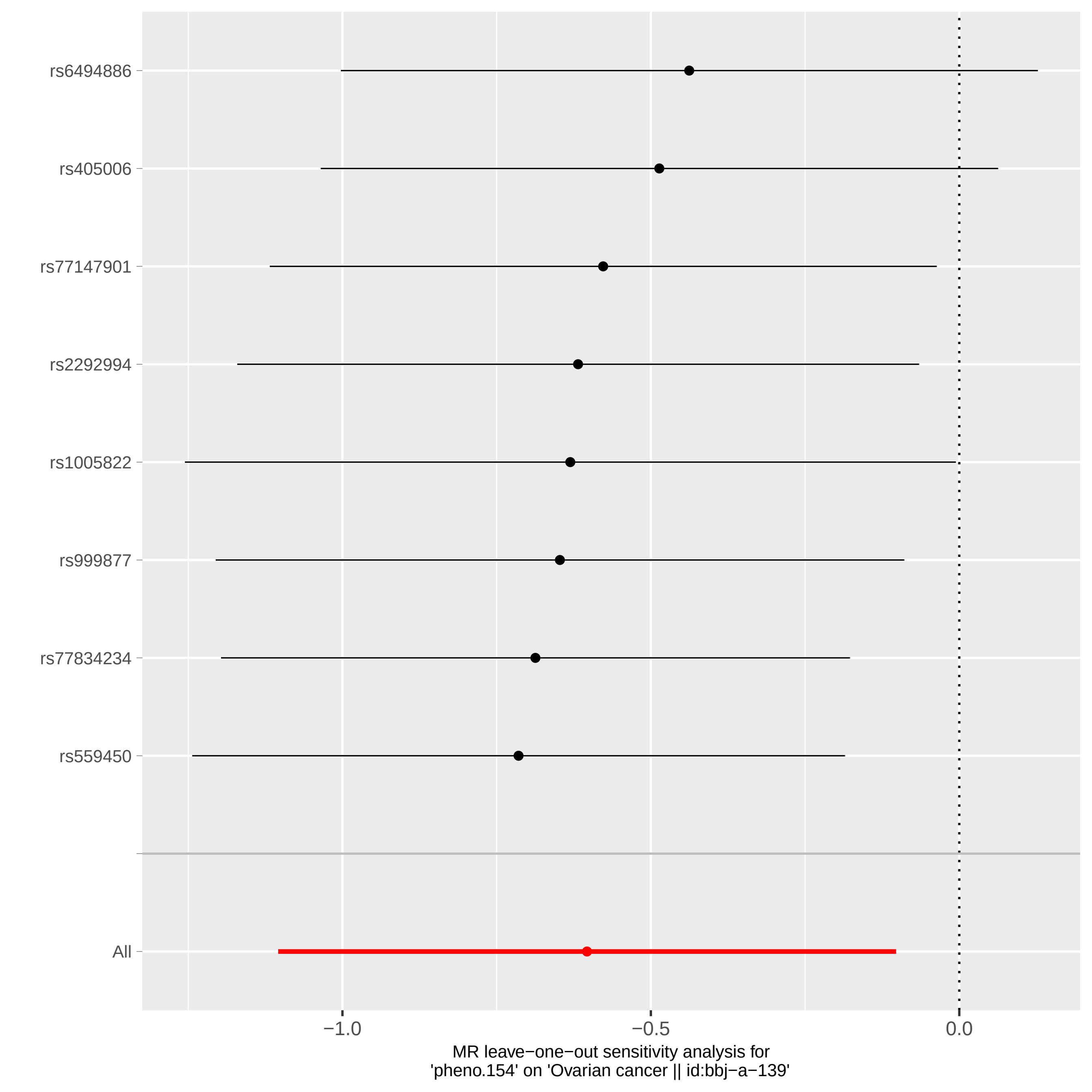
rs6494886
rs405006
rs77147901
rs2292994
rs1005822
rs999877
rs77834234
rs559450
All
−1.0
−0.5
0.0
MR leave−one−out sensitivity analysis for
'pheno.154' on 'Ovarian cancer || id:bbj−a−139'
